# Aberrant origin of the occipital artery from the internal carotid artery: utility of the occipital tap maneuver

**DOI:** 10.1016/j.jvscit.2021.09.013

**Published:** 2021-10-14

**Authors:** Enrico Premi, Andrea Pilotto, Alberto Benussi, Francesca Prandini, Mauro Magoni, Alessandro Padovani

**Affiliations:** a Stroke Unit, Department of Neurological and Vision Sciences, ASST Spedali Civili, Brescia, Italy; b Neurology Unit, Department of Neurological and Vision Sciences, ASST Spedali Civili, Brescia, Italy; c Neurology Unit, Department of Clinical and Experimental Sciences, University of Brescia, Brescia, Italy; d Neuroradiology Unit, University of Brescia, Brescia, Italy

**Keywords:** Ascending pharyngeal artery, Doppler ultrasound, Occipital artery, Occipital tap maneuver

## Abstract

We have described a case of a suspected transient ischemic attack with a double lumen potentially resembling a dissection of the internal carotid artery on Doppler ultrasound. The identification of an arterial branch from the internal carotid artery and flowing near it using magnetic resonance imaging suggested an aberrant origin of an ascending pharyngeal artery or occipital artery. Performance of the occipital tap maneuver in the occipital artery perfusion territory demonstrated a reflected flow in the double lumen, supporting the aberrant origin of the occipital artery from the internal carotid artery. The occipital tap maneuver can represent an easy-to-do procedure to distinguish anatomic variants and study double-lumen findings.

## Case report

**Fig 1:**
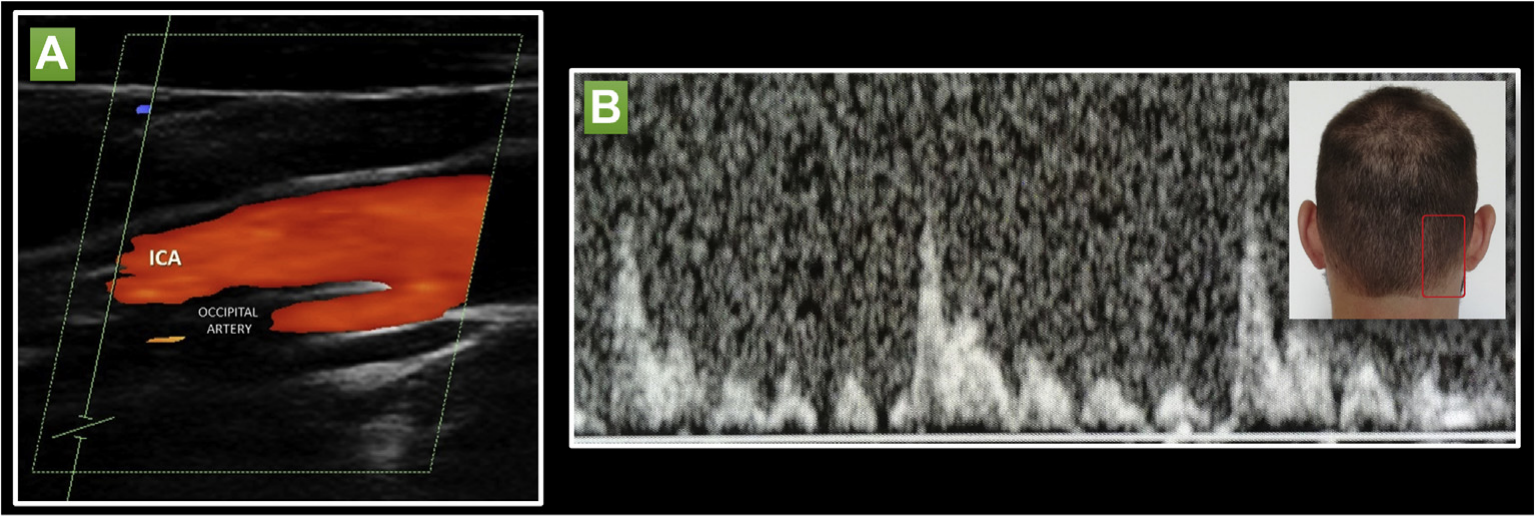
**A,** Carotid duplex ultrasound showing an internal right carotid artery double lumen. **B,** Occipital tap maneuver: during the assessment of the double lumen (occipital artery), tapping of the occipital artery produced a reflected oscillatory flow. A representative view of the tapping site in the occipital artery perfusion territory is shown (not a representation of the described patient).

**Fig 2:**
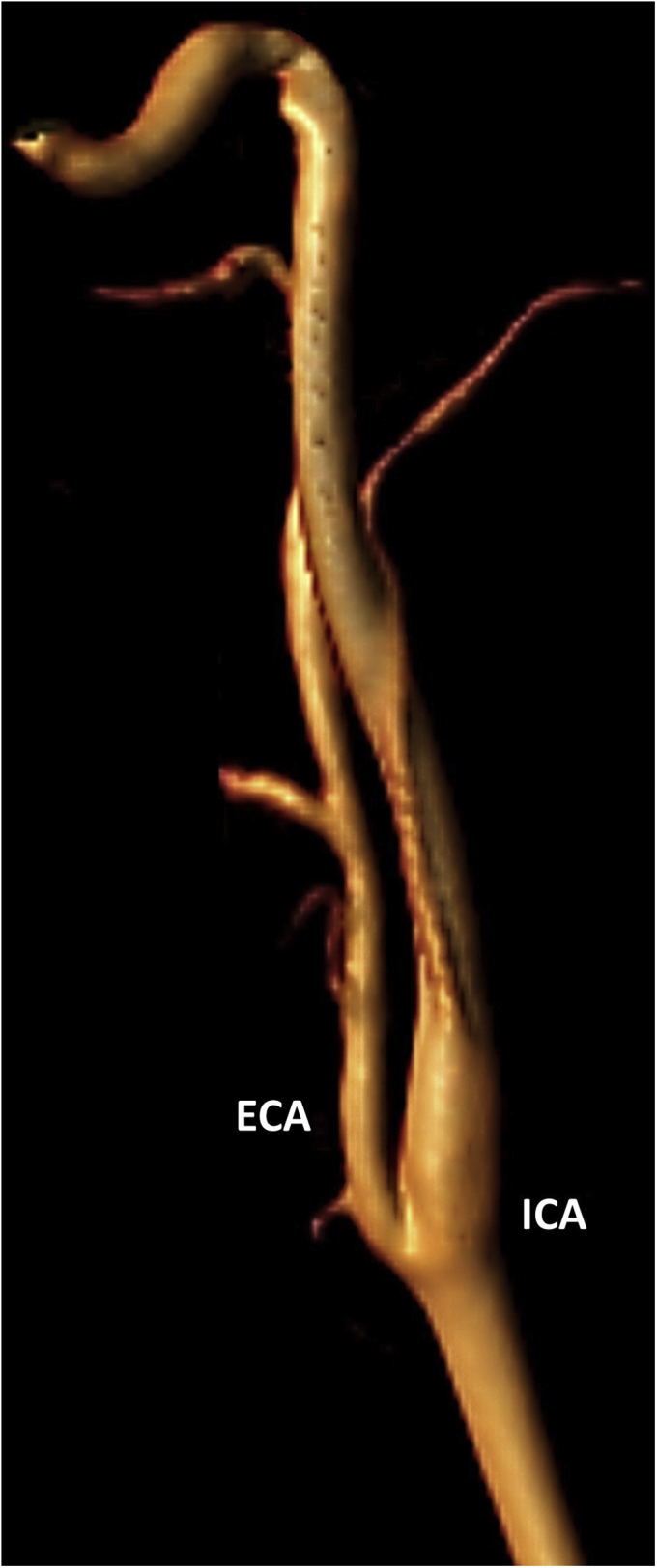
Magnetic resonance angiogram of the supra-aortic vessels showing the occipital artery originating from the internal carotid artery.

A 28-year-old woman was admitted to the neurology department after two transitory episodes lasting several hours of language impairment resembling motor aphasia and mild confusion. On admission, the neurologic examination, brain computed tomography scan, and electroencephalographic findings were unremarkable. A transient ischemic attack was suspected by the clinical symptoms and medical history. Thus, the patient underwent a carotid duplex ultrasound examination, which showed a parallel arterial flow at the origin of the internal carotid artery, potentially resembling a double lumen (Fig 1). To exclude the dissection of the internal carotid artery, considering the very close proximity of the double lumen to the internal carotid artery for an extensive length, magnetic resonance angiography of the neck vessels was performed. Magnetic resonance imaging excluded a carotid artery dissection but identified an arterial branch arising from the post–bulbar internal carotid artery and flowing in close proximity to it (Fig 2). The patient provided written informed consent for the report of her case details and imaging studies.

From a neurologic viewpoint, carotid duplex ultrasound is widely used in everyday practice, especially when a cerebrovascular etiology is suspected and for challenging conditions such as carotid dissection. Considering that normal ultrasound examination findings will not rule out a priori the possibility of a carotid dissection, the presence of supportive ultrasound findings (eg, a potential double lumen) should deserve great attention and further diagnostic examinations (eg, angiography with magnetic resonance imaging and/or computed tomography).^1^ In contrast, the evaluation of potential anatomic variants of the external carotid artery is a very rare eventuality in neurologic ultrasound examination of supra-aortic trunks.

**Fig 3:**
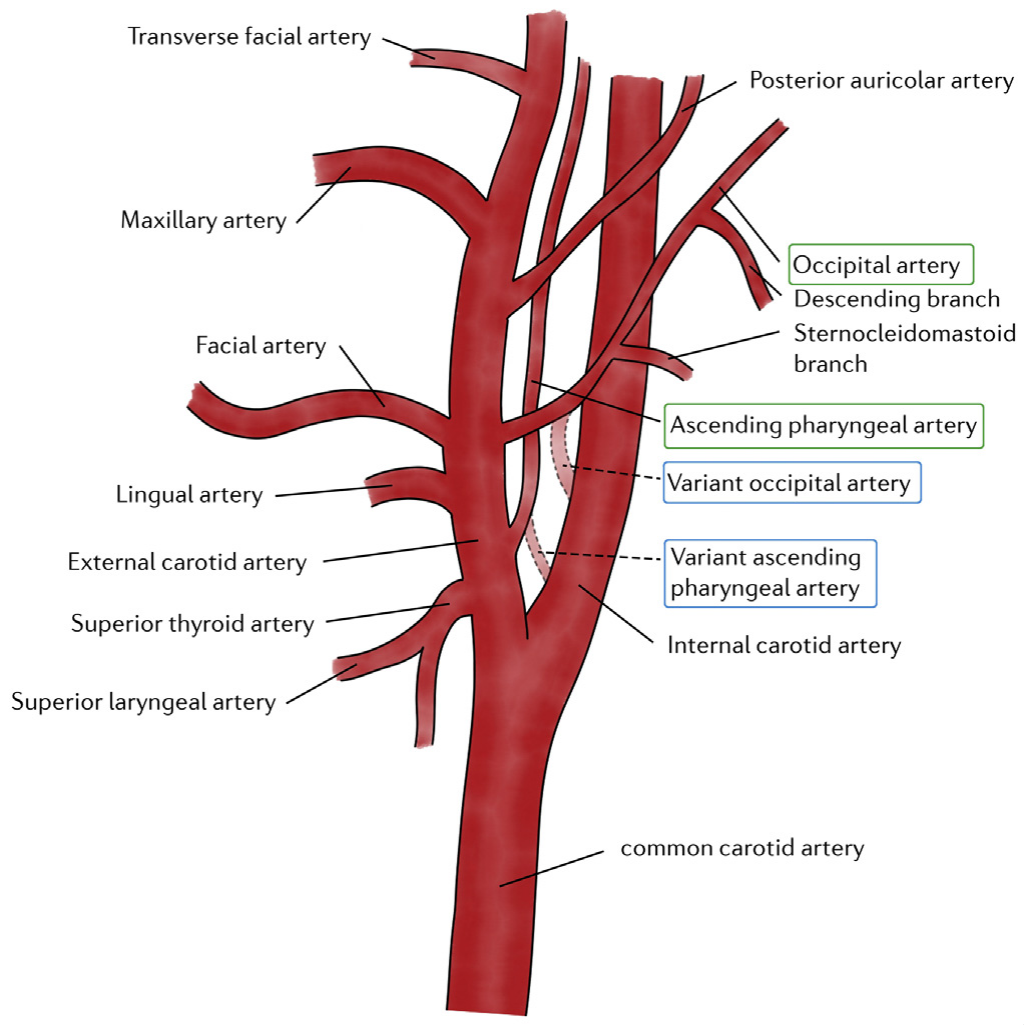
Drawing showing anatomic view of carotid bifurcation (internal and external carotid arteries). Canonical (*green*) from external carotid artery and aberrant (*light blue*) from internal carotid artery origins of ascending pharyngeal artery and occipital artery are also shown.

Therefore, considering the anatomy of the neck vessels, two anatomic variants with an aberrant origin should be considered: (1) an ascending pharyngeal artery^2^^,^^3^ or (2) an occipital artery.^4^^,^^5^ In both cases, these arteries will usually arise from the external carotid artery but can rarely present as anatomic variants with an aberrant origin from the internal carotid artery^2^, ^3^, ^4^, ^5^ (Fig 3). The temporal tap maneuver (with several limitations) is routinely applied to differentiate the external and internal carotid arteries (with tapping over the ipsilateral superficial temporal artery aiming to produce a reflected flow in the external carotid artery) during Doppler ultrasound examination of the carotid bifurcation).^6^^,^^7^

Taking inspiration from these factors, we performed an occipital tap maneuver over the right occipital pole in the perfusion territory of occipital artery (where it passes under the sternocleidomastoid muscle and then perforates the fascia to connect the cranial attachment of the trapezius with the sternocleidomastoid muscle; Fig 1). During Doppler ultrasound, a reflected flow in the double lumen was observed, potentially differentiating these two anatomic variants and confirming the aberrant origin of the occipital artery from the internal carotid artery (Fig 1; Supplementary Video). To the best of our knowledge, the present report is the first description of an anatomic variant (aberrant occipital artery) of the supra-aortic trunks potentially resembling a double-lumen appearance of the internal carotid artery.

## Conclusions

Although rare, this eventuality should be considered during Doppler ultrasound evaluation of this anatomic district. The occipital tap maneuver can represent an easy-to-do procedure that could help in distinguishing an aberrant origin of the pharyngeal or occipital artery and in supporting the evaluation of a double lumen when suspecting a potential dissection of the internal carotid artery.

## Appendix

Supplementary Video The video summarizes the ultrasound findings of the present report using B-mode imaging, color imaging (image and video), Doppler mode, occipital tap maneuver. For the tap maneuver, the anatomic localization of the tapping is demonstrated (**right panel;** highlighted in *red*). Arterial pulsations are highlighted with *red asterisks*. The representative tapping on the Doppler trace is highlighted with *green circles*.
